# British Hospital Progress—1887-1913

**Published:** 1914-02-28

**Authors:** 


### The Hospital

The Workers' Newspaper of Administrative Medicine and Institutional Life, Administration, National Insurance and Health.

No. 1444, Vol. LV. SATURDAY, FEBRUARY 28, 1914. PRICE ONE PENNY

### BRITISH HOSPITAL PROGRESS—1887-1913.

Looking back over twenty-five years, it is a pleasure to recall the Hospital Almanac, which was published in 1888 at the instigation of the then Chairman of the London Hospital, Mr. Carr Gomme, in order to bring together a terse account of the chief Metropolitan hospitals and of up-to-date matters which were of interest to all the managers. Mr. Carr Gomme gave material assistance in the compilation of the Almanac, and it is likely to have a historic interest, from the fact that it really constituted the first issue of the book now known as "Burdett's Hospitals and Charities." All those who have been associated with the voluntary hospitals during the years which have intervened, or who have a knowledge of what they were in the early 'eighties, cannot fail to be amazed and grateful whenever they find themselves in a large, up-to-date, modern hospital in this country, with the time to spend a few hours in going through each portion of it. In the 'eighties special departments were few, the numbers of the medical staff were strictly limited, the equipment was often far from perfect, and the operation theatre a spacious chamber with accommodation for at least two hundred people, and often for many more. The bare recital of these features of the principal hospitals of thirty years ago is enough to bring forcibly to the apprehension of the workers in hospitals to-day how momentous and wonderful have been the changes and progress which science and experience have introduced, during the evolution of the modern up-to-date hospital.

If we carefully survey the fields of hospital treatment and hospital administration, the changes, improvements, and results during these thirty years will prove to have been momentous. One instance may reveal at a glance the revolution in opinion, in treatment, and in the outlook now presented by hospital care to the patients suffering from certain maladies. Thirty years ago the Samaritan wards for both sexes were still a feature of most hospitals, and by no means an agreeable feature either. The inmates of these wards were regarded as outcasts—and small wonder if they sometimes behaved themselves as such—and the cases were often shocking from the severity of the symptoms. Gradually the practice grew up of not admitting venereal disease to the wards, and such cases as presented themselves were relegated to the out-patient department. From inquiries we have made, and our own observations over a period of years, we should say that the number of these cases to be dealt with in the present day is infinitely fewer than in the past, and that the average severity is much less even in the worst cases. Science has, however, steadily fought the battle of the patients who may fall victims to these diseases, and the latest voluntary hospital development is to establish special wards for selected cases, because the approved treatment of to-day needs such accommodation, whilst the results of treatment when earlier applied are so effective as to reduce in-patient care to a relatively short time. Fifteen or twenty years ago it would not have been possible to have set up a new department for these cases. To-day public opinion, thanks to science, recognises its necessity and justification, and no protest is raised. It is quite worth the while of anyone interested in our hospitals to get the report of a large hospital of thirty years ago, and to compare the pages on which the names of the various departments and the members of the medical staff are fully set forth. One glance at these reports will bring out graphically the widespread and extended changes and developments which have occurred in the classification and treatment of cases to-day, as compared with thirty years ago.

A bird's-eye view of a British, modern, up-to-date hospital under the voluntary system shows that it deserves to have the reliable record of its far-reaching results and splendid efficiency which is contained in the Book of the Hospitals.* This work, which has appeared year by year for twenty-five years, has succeeded in keeping pace with the best traditions and results of the work of the 6,000 institutions which find a place in its pages. Indeed, as the press has recorded, the edition of 1914, partially historical in character, should be obtained

* *Burdett's Hospitals and Charities*, 1914. Twenty-fifth year. (London : The Scientific Press, Ltd.)

and preserved by everyone interested in institutional work, whether it relates to hospitals or to any other section of the field of charity. The Book of the Hospitals for 1914, which embodies the figures relating to the work of all the institutions to December 31, 1912, being as it was ready for circulation in January this year, is an event welcomed by every intelligent hospital worker, to whose co-operation and interest the completeness which the book has attained is largely due. We could wish that members of hospital committees, as well as members of the working staff in the first rank, and all the younger men throughout the British hospital world, would take an opportunity during 1914 to look through the pages of some of the earlier volumes and compare them with that of 1914. The twenty-fifth volume of the Book of the Hospitals may fairly claim to be the completest and most reliable collection of hospital statistics, based on audited figures, which has ever been issued from the press.

